# Theory of MBE Growth of Nanowires on Reflecting Substrates

**DOI:** 10.3390/nano12020253

**Published:** 2022-01-14

**Authors:** Vladimir G. Dubrovskii

**Affiliations:** Faculty of Physics, St. Petersburg State University, Universitetskaya Emb. 13B, 199034 St. Petersburg, Russia; dubrovskii@mail.ioffe.ru

**Keywords:** III-V nanowires, molecular beam epitaxy, reflecting substrate, re-emission, shadowing, nanowire length and radius, modeling

## Abstract

Selective area growth (SAG) of III-V nanowires (NWs) by molecular beam epitaxy (MBE) and related epitaxy techniques offer several advantages over growth on unpatterned substrates. Here, an analytic model for the total flux of group III atoms impinging NWs is presented, which accounts for specular re-emission from the mask surface and the shadowing effect in the absence of surface diffusion from the substrate. An expression is given for the shadowing length of NWs corresponding to the full shadowing of the mask. Axial and radial NW growths are considered in different stages, including the stage of purely axial growth, intermediate stage with radial growth, and asymptotic stage, where the NWs receive the maximum flux determined by the array pitch. The model provides good fits with the data obtained for different vapor–liquid–solid and catalyst-free III-V NWs.

## 1. Introduction

Semiconductor NWs, particularly III-V NWs, are widely considered fundamental building blocks for nanoscience and nanotechnology and useful for applications in nanoelectronics and nanophotonics [1,2,3,4,5]. Very efficient elastic stress relaxation on strain-free NW side facets allows for dislocation-free growth in material systems with high lattice mismatch [6,7,8]. For example, planar growth of InAs on Si substrates (lattice mismatch = 11.6%) is difficult and leads to Volmer–Weber islands [9], which often contain crystallographic defects, while fully coherent growth of InAs NWs on Si is possible, provided that the NW diameter is smaller than critical (~25 nm [4]). A high aspect ratio (length over radius) is crucial for the applications and fundamental physical properties of NWs, such as one-dimensional transport of charge carriers, directional light emission, crystal purity within axial or radial heterostructures, abruptness of heterointerfaces, and crystal phase switching in III-V NWs [10]. These features explain the importance of controlling the NW dimensions during growth.

III-V NWs are often fabricated by MBE via metal-catalyzed vapor–liquid–solid (VLS) growth (with either Au [11,12,13,14,15,16] or group III [17,18,19] droplets) or catalyst-free SAG [20]. At low temperatures, NW growth transitions to the vapor–solid–solid (VSS) mode controlled by solid-phase diffusion through a frozen nanoparticle [21]. Catalyst-free SAG of NWs necessarily requires patterning of the substrate surface. SAG-MBE growth of VLS III-V NWs is achieved by preparation of Au seeds inside the pinholes in SiO_x_ [16] or SiN_x_ [22] mask layers or pre-deposition of a group III metal (usually, Ga or In) into the pinholes in SiO_x_/Si(111) for the self-catalyzed VLS process [18,19]. SAG of NWs offers several important advantages over growth on unpatterned substrates [12,13,14,15], including the absence of parasitic layer between the NWs [16] and, consequently, very low surface roughness, improved thermal conductivity in the substrate plane, regular positioning, and narrow size distributions of NWs in terms of both lengths and radii [10].

Due to the known high volatility of group V species such as As and P, their desorption from a catalyst nanoparticle and different NW facets occurs even at low temperatures. Surface diffusion of group V atoms is negligible [10]. Conversely, group III atoms may diffuse on different surfaces or incorporate with the NW sidewalls without desorption. The total balance of group III atoms is therefore more relevant for modeling NW growth [18]. There are two main mechanisms for material transport of group III atoms from the substrate surface to NWs. The first is surface diffusion of adatoms, which stick to the substrate and then diffuse to the NW base and along the sidewalls [23], as usually considered in modeling NW growth on unpatterned “sticky” substrates [12,13,14,16]. The second is re-emission of group III atoms from the reflecting substrate covered with an inert mask [17,18,22,24]. Several authors [16,22,24] attempted to model the growth kinetics of NW arrays grown by MBE on reflecting substrates using a cosine law (Lambert scattering) for re-emitted flux without rigorous justification. Furthermore, shadowing of both direct and re-emitted [16] or direct [16,22] fluxes was not taken into account. An important step forward was taken in Ref. [18], where Ga-catalyzed VLS growth of GaP NWs in patterned arrays of pinholes in SiO_2_/Si(111) was monitored using GaAsP markers. A model was developed that allowed for calculation of the total Ga flux into the NW (contributing to the axial and radial NW growths along with the droplet swelling), without any free parameters. One important conclusion was that the specular re-emission model gave the best fit to the data compared to the cosine law or random angular reflection. However, no analytical expression for the total flux was given, and no NW growth modeling was presented.

In this work, I try to fill the gap by developing an analytic model for the total group III flux influenced by specular re-emission and shadowing. This allows one to determine the shadowing length of NWs corresponding to the full shadowing of the substrate surface in MBE. Different stages of NW growth are considered, including purely axial growth, axial and radial growths after reaching the critical diffusion length, and the asymptotic growth stage, where each NW receives a maximum flux determined by the pitch of a regular array. Good fits with the data on the growth kinetics of Ga-catalyzed GaP NWs, Au-catalyzed InP NWs, and catalyst-free InAs NWs are obtained. The model is quite general and should work equally well for metal-catalyzed and catalyst-free NWs in different material systems, where MBE SAG is performed on a masked substrate.

## 2. Assumptions and Model Parameters

A full description of MBE growth of III-V NWs on reflecting substrates that accounts for all possible factors, influencing the growth process and NW morphology, is beyond reach to this end. Here, a simplified analytical model is developed using the following assumptions. First, an NW is approximated as a cylindrical rod with uniform radius R from base to top, neglecting all tapering effects. An approximately cylindrical shape is often observed in self-catalyzed VLS III-V NWs [17,18], some Au-catalyzed III-V NWs [13], and catalyst-free SAE III-V NWs [20]. However, more complex tapered or pencil-like shapes are also possible [14]. Second, MBE growth on rotating substrates is considered, where the particular geometry of an array of pinholes (for example, square or hexagonal) should not critically influence the NW growth process. Therefore, only the NW surface density N enters the results. It can be related to the surface area per NW $P^{2}$ as $N=P^{-2}$, where P is the pitch of the square array. The results can easily be re-formulated for any geometry (for example, using $P^{2}=(\sqrt{3}/2){\tilde{P}}^{2}$, where $\tilde{P}$ is the pitch of the hexagonal array [18]). Some works, for example, Refs. [18,24], explicitly take the array geometry into account, in which case deriving any analytical expression for the reflected flux is very difficult. Consequently, here we only study the dependence of the NW growth kinetics on N or P, assuming the influence of the array geometry as being a second-order effect. Third, an idealized ensemble of NWs having identical lengths L and radii R is considered. Fourth, the incorporation limited diffusion length of group III adatoms on the NW sidewalls, λ, is introduced [10]. With the neglect of evaporation of group III adatoms from the NW side facets, surface incorporation leads to radial growth.

In the absence of desorption of group III atoms from the NW sidewalls and top (with or without a catalyst nanoparticle), the total balance of group III atoms is given by $v=N{\left(dV/dt\right)}_{dir}+v_{ref}$. Here, v is the direct group III flux onto the surface (nm/s), $v_{ref}$ is the group III flux reflected from the substrate, V is the NW volume including the nanoparticle resting on its top for VLS or VSS growth process, and ${\left(dV/dt\right)}_{dir}=F_{dir}$, with $F_{dir}$ as the direct group III atomic current onto the NW (nm^3^/s). When the substrate surface is entirely blocked by NWs due to the shadowing effect in the directional MBE technique, the reflected flux becomes zero. Starting from this moment of time, the total balance of group III atoms gives $F_{dir}=F_{max}=v/N=vP^{2}$ (at $v_{ref}=0$), as in Ref. [18], meaning that each NW receives the maximum flux$\;F_{max}=vP^{2}$, which equals the group III flux times the surface area per one NW $P^{2}$. Thus, the variables considered are the time-dependent NW length L(t) and radius R(t), with the control parameters P, λ, v, and the initial NW radius $R_{0}$.

## 3. Total Material Flux into the Nanowire

**Figure 1:**
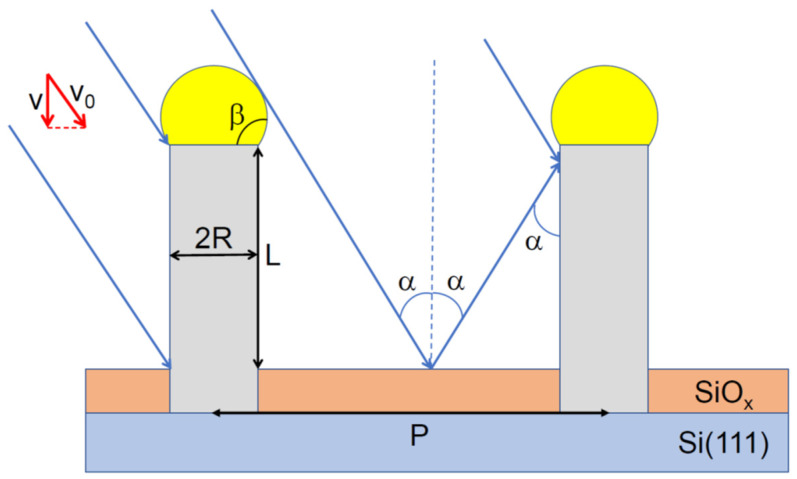
Illustration of NW growth by selective area VLS-MBE on a reflecting substrate in the case of specular reflection, where group III atoms simply bounce off the silica mask. P denotes the pitch of the NW array. NWs are considered cylinders with length L above the mask surface and uniform radius R. The reflected flux impinges the NW side facets at the same angle α as the primary flux.

MBE growth of NWs on a reflecting substrate is illustrated in Figure 1. The group III flux onto the substrate surface equals $v=v_{0}cos\alpha$, with $v_{0}$ as the total group III flux and α as the beam angle with respect to the substrate normal. The nominal thickness of planar material deposited by the moment of time t equals H=vt. I take into account re-emission (reflection) of group III atoms from the mask surface and shadowing of the mask by the NW array. Re-emission from the NW sidewalls or catalyst nanoparticles is neglected. Hence, any material exchange between the NWs [25,26] is not considered. Surface growth on the mask is assumed negligible. As mentioned above, all NWs in the array are considered identical in terms of their length and radius and modeled as cylinders with length *L* above the mask surface and uniform radius *R*.

The total current into the NW, $F=F_{dir}+F_{ref}$, contains contributions from the direct ($F_{dir}$) and reflected ($F_{ref}$) fluxes. The maximum current is given by $F_{max}=vP^{2}$, as discussed above. The direct current is given by$\;F_{dir}=vS$ if $S/P^{2}<1\;\mathrm{and}\;vP^{2}\;\mathrm{if}\;S/P^{2}\geq 1,$ where S is the NW surface area (including the nanoparticle surface area for metal-catalyzed NWs or NW top facet for catalyst-free SAE NWs) exposed to the direct flux. The expression $F_{dir}=vS$ is the common definition of the direct material flux onto the NW [10,18,27,28]. It shows that the volume of group III atoms impinging the NW per unit time equals the group III flux times the total surface area of this NW exposed to the flux. However, this expression does not account for the shadowing effect. When S becomes larger than $P^{2}$, the direct flux can no longer increase. Instead, it stabilizes at the maximum value $vP^{2}$, meaning that each NW in the array of N NWs per unit surface area receives a part of the total flux $vP^{2}=v/N$.

Similarly, the reflected current impinging the NW is $F_{ref}=v_{ref}S^{\prime }$, where $S^{\prime }$ is the NW surface area (including its top) exposed to the reflected flux. From the total balance of group III atoms, it follows that $v_{ref}=v(1-S/P^{2})\;\mathrm{if}\;S/P^{2}<1$ and 0 if $S/P^{2}\geq 1$. These considerations give the total flux in the form

(1) $$F=v\left[S+\left(1-\frac{S}{P^{2}}\right)S^{\prime }\right],\;\frac{S}{P^{2}}<1,\;F=vP^{2},\;\frac{S}{P^{2}}\geq 1.$$

For the total flux normalized to its maximum value $F_{max}$, Equation (1) takes the form

(2) $$\frac{F}{F_{max}}=\frac{S}{P^{2}}+\left(1-\frac{S}{P^{2}}\right)\frac{S^{\prime }}{P^{2}},\frac{S}{P^{2}}<1,\;\frac{F}{F_{max}}=1,\;\frac{S}{P^{2}}\geq 1.$$

For VLS NWs, the total group III flux equals the derivative of the NW volume plus the droplet volume with respect to time

(3) $$F=\frac{d}{dt}\left[\pi R^{2}L+\frac{\pi R^{3}}{3}f\left(\beta \right)\right]$$

Here, f(β)=(1−cosβ)(2+cosβ)/[(1+cosβ)sinβ] is the known geometrical function of the droplet contact angle β [6]. For catalyst-free SAG NWs, f(β)=0, and only the first term remains on the right side of Equation (3). This model for the total flux is purely geometrical, as in some previous works on NW growth modeling [14,27], and captures the two major effects: (i) re-emission from the mask and (ii) shadowing of the mask surface, which finally leads to a given maximum flux per NW determined by the array pitch [28].

The above expressions are insensitive to the re-emission law. In the case of specular reflection, where group III atoms just bounce off the mask surface [18], the surface areas exposed to the direct and reflected fluxes are given by

(4) $$S=2RLtan\alpha +\frac{\chi }{cos\alpha }\pi R^{2},\;S^{\prime }=2RLtan\alpha +\frac{\chi^{\prime }}{cos\alpha }\pi R^{2}.$$

Here, the first terms stand for the surface area of NW sidewalls exposed to the flux and are identical for the direct and reflected fluxes in the case of specular re-emission. The droplet surface areas intercepted by the fluxes contain different geometrical coefficients χ and$\;\chi^{\prime }$, which can be obtained using the approach of Ref. [29] as functions of the two angles α and β. In the case of catalyst-free SAG NWs, one simply has $\chi =cos\alpha \;\mathrm{and}\;\chi^{\prime }=0$, meaning that the flux impinging the flat top surface is the same as for the substrate surface and no reflected atoms can impinge the NW top.

**Figure 2:**
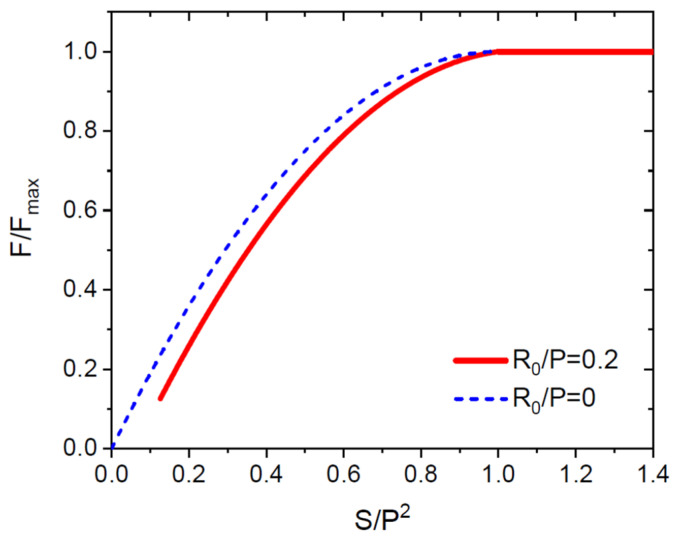
Normalized group III atomic current versus the normalized NW surface area $x=S/P^{2}$ obtained from Equation (5) for catalyst-free NWs at a constant radius $R_{0}\;$for $R_{0}/P=0.2$ and $R_{0}/P\to 0$. In the latter case, the dependence is reduced to $F/F_{max}=2x-x^{2}$.

Introducing $x=S/P^{2}$, Equation (2) can be put in the dimensionless form

(5) $$\begin{array}{l} \frac{F}{F_{max}}=x+\left(1-x\right)\left(x-\frac{\left(\chi -\chi^{\prime }\right)}{cos\alpha }\frac{\pi R^{2}}{P^{2}}\right)≅2x-x^{2},\;x<1,\\ \frac{F}{F_{max}}=1,\;x\geq 1, \end{array}$$

with S given by Equation (4). This dependence is shown in Figure 2 for catalyst-free SAG at a constant NW radius $R=R_{0}=0.2P$. The dashed curve shows the approximation $F/F_{max}=2x-x^{2}$, which becomes more accurate for NWs with higher aspect ratios. It clearly shows that the flux impinging the NW side facets equals twice the direct flux at the beginning of growth due to re-emission and becomes $F_{max}$ at x=1 due to shadowing.

Full shadowing of the mask surface occurs at $S=S_{*}=P^{2}$. From Equation (4), one obtains the shadowing length in the form

(6) $$L_{*}=\frac{cotan\alpha }{2R_{*}}P^{2}-\frac{\chi_{*}\pi }{2sin\alpha }R_{*}$$

**Figure 3:**
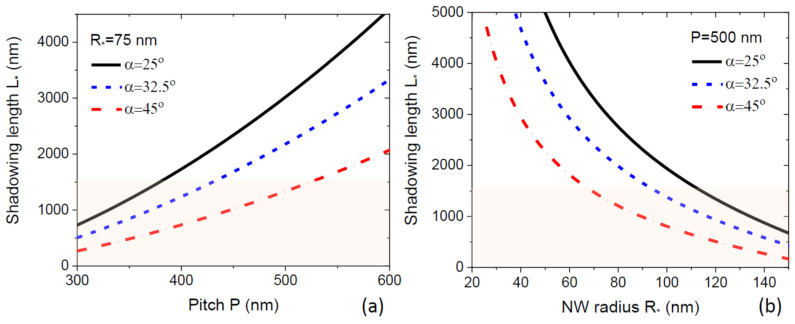
Shadowing length versus (**a**) the array pitch at a fixed NW radius of 75 nm and (**b**) NW radius at a fixed pitch of 500 nm for three different beam angles shown in the legend. Shadowing length increases with the pitch and decreases with the radius and the beam angle. The colored zones correspond to the absence of radial growth ($R_{*}=R_{0}$) for GaAs NWs at a diffusion length of Ga adatoms of 1500 nm.

Here, $R_{*}$ is the NW radius and $\chi_{*}=\chi \left(\alpha ,\beta_{*}\right)$ is the geometrical function of the droplet angle $\beta_{*}$ reached at the moment of time where $S=S_{*}$. This $L_{*}$ depends on the NW radius because thicker NWs shadow a larger surface area, and hence the full shadowing of the mask occurs at a shorter NW length. The shadowing length is shown in Figure 3 as a function of the pitch (a) and radius (b) for different beam angles, at a fixed $\beta_{*}$ of 135°, corresponding to $\chi_{*}=1/sin^{2}\beta_{*}=$ 2.0. As expected, the shadowing length increases with the pitch and decreases for thicker NWs and larger beam angles. The radial NW growth will never occur $\mathrm{when}L_{*}\left(R_{0}\right)<\lambda$ , with λ being the diffusion length of group III adatoms on the NW sidewalls, limited by surface incorporation in the absence of desorption, as discussed above. In this case, group III atoms are collected from the top part of the NW of height $L_{*}\left(R_{0}\right)$, while the rest of the NW is shadowed. All these atoms will diffuse to the NW top due to $L_{*}\left(R_{0}\right)<\lambda$, and none of them will incorporate with the NW sidewalls. The published estimates for the Ga diffusion length on (110) side facets of <111>-oriented GaAs NWs equal 1500 nm [14], 1800 nm [30], and more than 2000 nm [13]. The shaded zones in Figure 3 correspond to the range of pitches and NW radii, where no radial growth occurs at λ=1500 nm.

It is well known that fabrication of thin III-V NWs by MBE is difficult, partly due to the radial growth [10]. According to our results, the radial growth can be more easily suppressed in dense arrays of NWs corresponding to smaller pitches and in MBE systems with larger beam angles α for group III atoms. It is desirable to ensure the condition $L_{*}\left(R_{0}\right)<\lambda$, where the NW radius $R_{0}\;$should be uniform from base to top and defined by the size of the growth seeds.

## 4. Evolution of Nanowire Length at a Constant Radius

The governing equation for the NW growth kinetics is given by

(7) $$\frac{d}{dH}\left[\pi R^{2}L+\frac{\pi R^{3}}{3}f\left(\beta \right)\right]=S+\left(1-\frac{S}{P^{2}}\right)S^{\prime }$$

with S and $S^{\prime }$ determined by Equation (4). Below, I will use

(8) $$\frac{d}{dH}\left[\frac{\pi R^{3}}{3}f\left(\beta \right)\right]=0$$

Meaning that the droplet volume remains constant during growth. This result is exact for catalyst-free SAG NWs, where the droplet volume is zero. It may also be justified for Au-catalyzed VLS NWs [10,12,13,15], in which case there is always a steady-state solution for a time-independent droplet volume, regulated by chemical potential of group V atoms in an Au-III-V droplet. However, Equation (8) is only approximate for self-catalyzed VLS NWs, where a group III droplet may swell or shrink depending on the atomic V/III ratio [18,31,32]. In the case of droplet swelling under excessive Ga flux, subsequent radial growth by step flow starting from the NW top leads to enlargement of the NW radius [18]. In any case, the ratio of the droplet volume over the NW volume scales as R/L, and hence the approximation given by Equation (8) is justified for long enough NWs having high aspect ratios.

Using Equations (4), (7), and (8) at $R=R_{0}$, one arrives at

(9) $$\frac{dL}{dH}=\frac{2Ltan\alpha }{\pi R_{0}}+\frac{\chi }{cos\alpha }+\left[1-\frac{\pi R_{0}^{2}}{P^{2}}\left(\frac{2Ltan\alpha }{\pi R_{0}}+\frac{\chi }{cos\alpha }\right)\right]\left(\frac{2Ltan\alpha }{\pi R_{0}}+\frac{\chi^{\prime }}{cos\alpha }\right),\;L\left(H=0\right)=0.\;$$

The exact solution for the NW length is given by

(10) $$L=\frac{\pi R_{0}cotan\alpha }{2}\left(\frac{y_{1}e^{H/H_{c}}+\omega y_{2}}{e^{H/H_{c}}+\omega }-\frac{\chi }{cos\alpha }\right),\;L<L_{*},\;$$

with coefficients

(11) $$y_{1}=\frac{B+\sqrt{B^{2}-4C}}{2},\;y_{2}=\frac{B-\sqrt{B^{2}-4C}}{2},\;B=\frac{2P^{2}}{\pi R_{0}^{2}}+\frac{\chi -\chi^{\prime }}{cos\alpha },\;C=\frac{\left(\chi -\chi^{\prime }\right)}{cos\alpha }\frac{P^{2}}{\pi R_{0}^{2\;}},\;\omega =\frac{y_{1}-\chi /cos\alpha }{\chi /cos\alpha -y_{2}},\;H_{c}=\frac{P^{2}cotan\alpha }{2R_{0}\sqrt{B^{2}-4C}}.$$

**Figure 4:**
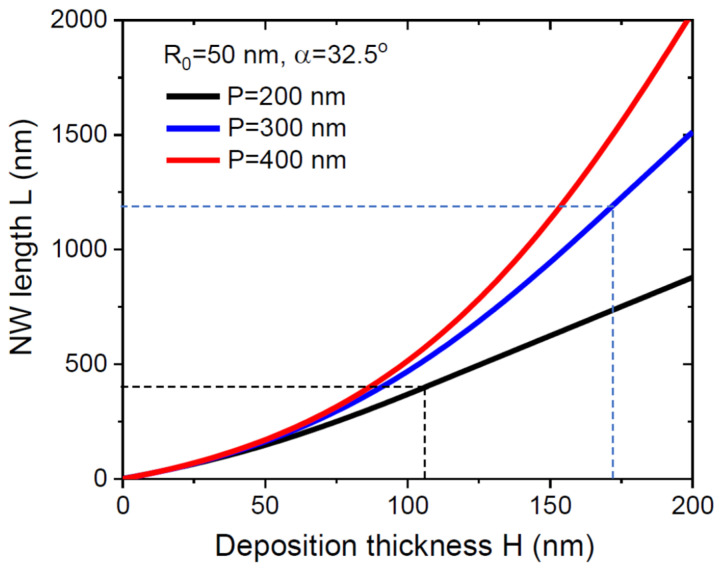
Evolution of the NW length with deposition thickness at a fixed NW radius of 50 nm for α= 32.5° and three different pitches shown in the legend. The curves are obtained from Equations (11)–(13) using the parameters summarized in Table 1. NW length increases with the pitch for a given H. Fast elongation in the initial stage is followed by a linear increase after the full shadowing of the mask surface, which occurs later for larger pitches. Dashed markers show the corresponding $H_{*}$ and $L_{*}$ for P= 200 and 300 nm. The shadowing length is larger than 2000 nm for P= 400 nm.

The NW elongation after the full shadowing of the substrate surface is given by

(12) $$L=L_{*}+\frac{P^{2}}{\pi R_{0}^{2}}\left(H-H_{*}\right),\;L\geq L_{*}$$

where $H_{*}\;$is the deposition thickness at which $L=L_{*}$. Figure 4 shows the evolution of the NW length with deposition thickness at a fixed NW radius of 50 nm, a beam angle of 32.5°, and a droplet contact angle of 125° for three different pitches of 200 nm, 300 nm, and 400 nm. The corresponding growth parameters are summarized in Table 1. As expected, the NWs elongate faster for larger pitches. It is noteworthy that $y_{1}\gg y_{2}$ in most cases, corresponding to a pitch-independent characteristic thickness $H_{c}≅\left(\pi /4\right)R_{0}cotan\alpha$, which does not change significantly with the pitch according to Table 1. Overall, the NWs elongate faster at the beginning of growth, while the length evolution becomes linear in H $\mathrm{at}\;L\geq L_{*}$. The initial growth stage is, however, more complex than the exponential increase in the NW length with time. The exponential growth stage was theoretically predicted and experimentally observed for NW growth on sticky substrates [13,14,33,34,35] and actually regardless of the epitaxy technique (directional MBE method or vapor-phase epitaxy). This important difference stems from the fact that the number of group III atoms collected by the NW sidewalls in MBE on sticky substrates is proportional to L, while in our case, it scales as 2L at the beginning and gradually decreases to L in the course of growth. In both cases, the group III flux converges to $F_{max}$ due to shadowing, but NWs on a reflecting substrate grow faster before that.

## 5. Radial Growth in the Intermediate Growth Stage

The case of $L_{*}>\lambda$ corresponds to the most complex scenario of the NW growth kinetics and morphology evolution, even in the case of cylindrical NW geometry without tapering. The radial growth starts before the full shadowing of the mask (when L reaches λ), where the total influx of group III atoms continues to increase. The moment of time where L = $\lambda \;\mathrm{relates}\;\mathrm{to}\;a\;\mathrm{deposition}\;\mathrm{thickness}\;H_{0}$, with $R\left(H=H_{0}\right)=R_{0}$. Further evolution of the NW volume is given by

(13) $$\frac{d}{dH}\left(\pi R^{2}L\right)=S+\left(1-\frac{S}{P^{2}}\right)S^{\prime }$$

assuming again a time-independent droplet volume. This equation is insufficient for finding L and R separately, which is why a second equation for L is needed. Using similar considerations as presented in Section 2 for the upper part of an NW of height λ, the evolution of the NW length is given by

(14) $$\frac{dL}{dH}=\frac{\chi }{cos\alpha }+\frac{2tan\alpha }{\pi }\frac{\lambda }{R}+\left(1-\frac{S}{P^{2}}\right)\left(\frac{\chi^{\prime }}{cos\alpha }+\frac{2tan\alpha }{\pi }\frac{\lambda }{R}\right)$$

Here, the first term gives the direct flux impinging the NW top section [13,14], while the second term describes the contribution from re-emitted group III atoms. From Equations (13) and (14), the NW radius evolves according to

(15) $$\frac{dR}{dH}=\frac{tan\alpha }{\pi }\left(1-\frac{\lambda }{L}\right)\left(2-\frac{S}{P^{2}}\right)$$

These two equations can be resolved only numerically.

However, with neglect of the contribution from the flux impinging the droplet or the top NW facet, Equation (14) simplifies to

(16) $$\frac{dL}{dH}≅\frac{2tan\alpha }{\pi }\frac{\lambda }{R}\left(2-\frac{S}{P^{2}}\right)$$

From Equations (15) and (16), the evolution of NW radius with its length is given by

(17) $$\frac{dR}{dL}=\frac{R}{2\lambda }\left(1-\frac{\lambda }{L}\right)$$

This equation has the solution

(18) $$R=R_{0}{\left(\frac{\lambda }{L}\right)}^{1/2}e^{\left(L/\lambda -1\right)/2}$$

Therefore, the NW volume scales exponentially with its length

(19) $$\pi R^{2}L=\pi R_{0}^{2}\;\lambda e^{L/\lambda -1}$$

This relationship contains only one fitting parameter λ and is independent of the re-emission and shadowing. The latter are described by the $\left(2-S/P^{2}\right)$ factors in Equations (15) and (16) but cancel in Equation (17) for dR/dL. Exponential dependence is expected to be more accurate for higher aspect ratio NWs and at larger diffusion length λ, corresponding to an almost negligible contribution of the NW top into the total collection area for group III atoms impinging the NW.

## 6. Asymptotic Growth Stage

This late stage of NW growth occurs for $S\geq P^{2}$, where the substrate surface is entirely shadowed by the NW array and hence is exactly identical for MBE growth of NWs on any substrate. The condition of a time-independent droplet volume becomes almost exact because the aspect ratio L/R increases in the course of growth. The governing equations are reduced to

(20) $$\frac{d}{dH}\left(\pi R^{2}L\right)=P^{2}$$

(21) $$\frac{dL}{dH}=\frac{\chi }{cos\alpha }+\frac{2tan\alpha }{\pi }\frac{\lambda }{R}$$

with the initial conditions $R\left(H=H_{*}\right)=R_{*}$ and $L\left(H=H_{*}\right)=L_{*}$. Integrating Equation (20), the NW radius is obtained in the form

(22) $$R=P{\left(\frac{h}{\pi L}\right)}^{1/2},\;h=H-H_{*}+h_{*},\;h_{*}=\frac{\pi R_{*}^{2}L_{*}}{P^{2}}.$$

Substitution of Equation (22) into Equation (21) yields

(23) $$\frac{dL}{dh}=\frac{\chi }{cos\alpha }+\varepsilon {\left(\frac{L}{h}\right)}^{1/2},\;\varepsilon =\frac{2tan\alpha }{\sqrt{\pi }}\frac{\lambda }{P}.$$

This is a special type of the Chini equation considered recently in Ref. [36], where it was used from the beginning of growth, assuming that all diffusive group III adatoms are collected by NWs from a “sticky” substrate surface. In our case, the solution is given by

(24) $$\frac{h}{h_{*}}=\frac{F\left(L/h\right)}{F\left(L_{*}/h_{*}\right)},\;F\left(L/h\right)=\frac{1}{L/h-\varepsilon \sqrt{L/h}-\chi /cos\alpha }{\left(\frac{2\sqrt{L/h}+\sqrt{\varepsilon^{2}+4\chi /cos\alpha }-\varepsilon }{2\sqrt{L/h}-\sqrt{\varepsilon^{2}+4\chi /cos\alpha }-\varepsilon }\right)}^{\frac{\varepsilon }{\sqrt{\varepsilon^{2}+4\chi /cos\alpha }}},$$

and is controlled by the two parameters χ/cosα and ε. The latter is inversely proportional to the array pitch, and hence the NW length should finally decrease with the pitch, the trend, which is inversed with respect to the growth start (see Figure 4).

In the large time limit, the L/H ratio and the radius saturate to the constants

(25) $$\frac{L}{H}\to \frac{1}{4}{\left(\sqrt{\varepsilon^{2}+4\chi /cos\alpha }+\varepsilon \right)}^{2},\;R\to \frac{2}{\sqrt{\varepsilon^{2}+4\chi }/cos\alpha +\varepsilon }\frac{P}{\sqrt{\pi }}.$$

Therefore, the NW length scales linearly with the deposition thickness and increases for larger ε and χ. The maximum possible NW radius $P/\sqrt{\pi }$ corresponds to coalescence of the NW array into continuous film. According to Equation (25), the steady-state radius decreases for larger ε and χ. The NWs will coalesce only at ε=0 (the absence of surface diffusion from the NW sidewalls to the top) and χ/cosα=1, as in catalyst-free SAG NWs. Whenever ε>0, surface diffusion of group III adatoms does not allow for coalescence of NWs into continuous film, that is, the NW length always remains larger than H and the radius smaller than $P/\sqrt{\pi }$.

**Figure 5:**
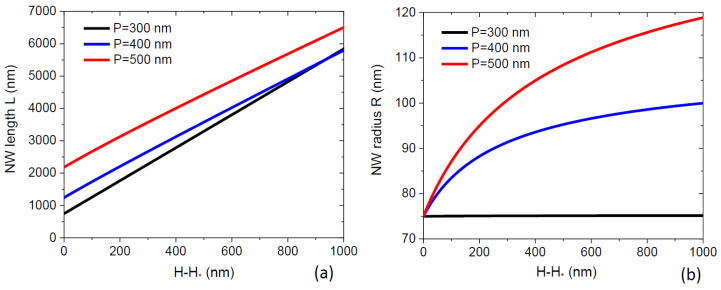
(**a**) NW length and (**b**) radius versus $H-H_{*}\;\;\mathrm{in}\;\mathrm{the}\;\mathrm{case}\;H>H_{*,}$ i.e., in the growth stage where the substrate surface is entirely shadowed by the NW array, obtained from Equations (22) and (25) for different pitches shown in the legend, assuming that all NWs had the same radius of 75 nm at $H=H_{*}.\;$The length increases linearly with the deposition thickness at P=300 nm, at an almost constant radius. The length curves become more sublinear with increasing the pitch, corresponding to the larger increase of the radii.

Figure 5 show the evolution of the NW lengths and radii, obtained from Equations (22) and (24) at a fixed α=32.5°, β=135° (χ=2.0) $R_{*}=$ 75 nm, λ= 500 nm, and three different pitches P= 300 nm, 400 nm, and 500 nm. The corresponding growth parameters are summarized in Table 2. For the smallest pitch of 300 nm, the radius stays almost constant, meaning that its steady-state limit has been reached in the earlier growth steps. At a constant radius, the NW length scales linearly with $H-H_{*}$. Increasing the pitch leads to a more pronounced radial growth such that the steady-state NW radius enlarges to 110 nm for P= 400 nm and 145 nm for P= 500 nm. As a consequence, the NW lengths increase more sublinearly with the deposition thickness. In the final step, the length of NWs grown in the 400 nm pitch array becomes smaller than that in the 300 nm pitch array. Such an inverse pitch dependence of the NW length was observed experimentally in Ref. [37].

## 7. Theory and Experiment

Self-catalyzed MBE growth of GaP NWs of Ref. [18] was performed at 600 °C using the SAG approach in a regular hexagonal array of patterned holes in a SiO_2_ mask layer on Si(111) ($P^{2}=$ 216,506 nm^2^), with α= 32.5° and v= 0.135 nm/s. Therefore, 60 min growth corresponded to $H_{max}=$ 486 nm. In total, 122 GaAsP markers were introduced at fixed time intervals to monitor ex situ the axial growth and radial extension of individual NWs. This linear dependence is used to convert the marker number to the Ga deposition thickness. The droplet contact angle stayed nearly constant at β= 135°, corresponding to the region of zincblende NWs, where the droplet volume increases by enlarging the base radius [38,39,40]. An almost untapered NW geometry was kept by step flow radial growth starting from the NW top [18]. In 60 min growth, the NW radius enlarged from 20 to 75 nm, approximately following the parabolic dependence $R\left(H\right)=20+0.161\times H-0.00012\times H^{2}$ nm. The NW axial growth rate increased linearly with H from 0.3 nm/s to 1 nm/s at H= 105.3 nm and stayed constant at 1 nm/s until the end of growth. Therefore, I use $L\left(H\right)=2.222\times H+0.02396\times H^{2}$ nm for 0≤H≤105.3 nm and L(H)=L(105.3 nm)+7.407×(H−105.3 nm) for H> 105.3 nm.

**Figure 6:**
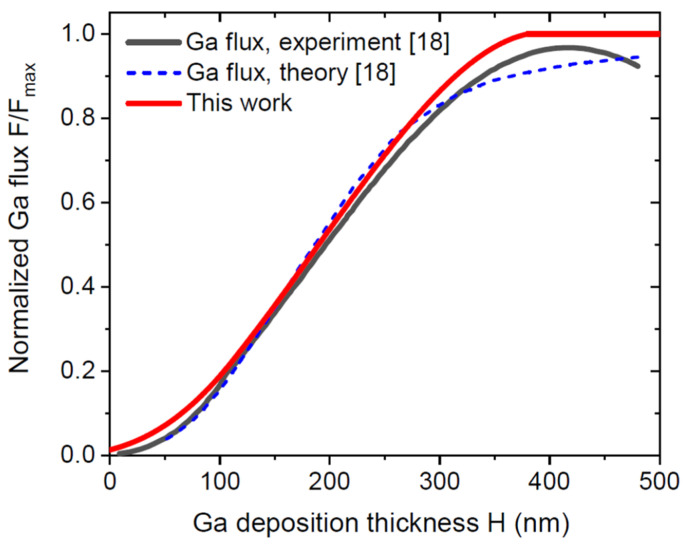
Normalized Ga current versus Ga deposition thickness. The bold black curve corresponds to the data of Ref. [18], obtained by monitoring the axial growth, radial extension, and increase in the Ga droplet volume in a self-catalyzed VLS GaP NW. The dashed blue line shows the modeling results of Ref. [18]. The bold red line is given by Equations (2) and (4) at a fixed β  of 135° using the dependences of the NW length and radius on H described in the main text.

With these R(H) and L(H), Equations (2) and (4) provide the fit to the measured Ga flux shown in Figure 6, which works equally well compared to a dedicated model of Ref. [18]. The discrepancy can be seen only for the highest Ga fluxes measured, where the model of Ref. [18] is not perfect either. Overall, our model predicts the maximum flux being reached at $S=P^{2}$, similarly to the model of Ref. [18], while the measured flux seems to decrease at the end of growth. The reason for this mismatch should be considered more carefully. One possible explanation is the partial evaporation of Ga atoms from the NW tip or the droplet in the late stage of growth, which is forbidden in our model.

**Figure 7:**
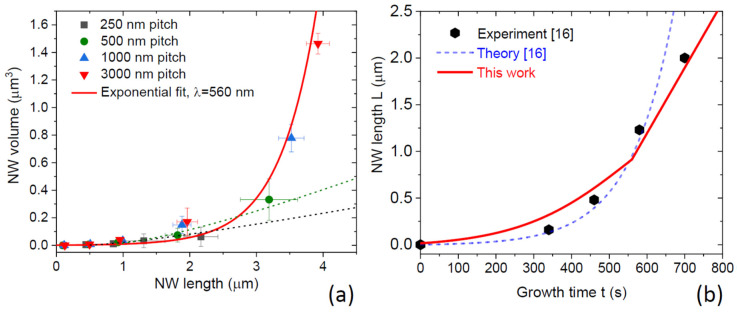
(**a**) NW volume versus length for catalyst-free SAG InAs NWs grown by MBE at 480 °C in regular square arrays of patterned holes in SiO_2_/Si(111). Symbols represent the data of Ref. [20]; NW radii at different data points are summarized in Table 3. Bold line shows exponential fit obtained from Equation (19) with λ= 560 nm. Dashed lines show the curves obtained with the shadowing effect for P= 250 nm and 500 nm. (**b**) Length of an Au-catalyzed, 12 nm radius InP NW versus the growth time: symbols represent the data of Ref. [16], dashed line shows the modeling results [16], bold line is the fit obtained from Equations (10) to (13) with P= 200 nm.

Catalyst-free InAs NWs of Ref. [20] were grown by SAG-MBE in patterned arrays of pinholes in SiO_2_/Si(111) at 480 °C. Square arrays with pitches P ranging from 250 to 3000 nm were investigated by measuring the average values of the NW lengths and diameters grown under identical conditions for different times. The data for the average values of NW lengths and diameters are given in Table 3, with the corresponding error bars shown in Figure 7a. Both lengths and diameters increased sublinearly with time [20]. However, if one plots the NW volume versus length, the exponential dependence fits all the datapoints within the error bars. The best fit is obtained using Equation (19) with λ= 560 nm and $R_{0}=$ 50 nm, as shown in Figure 7a. Including the shadowing effect, which is important for the smallest pitches, leads to the curves shown by the dashed lines in Figure 7a.

In Ref. [16], SAG of Au-catalyzed InP NWs was performed by chemical beam epitaxy (another directional deposition method that is similar to MBE in this respect) in hexagonal arrays of patterned holes in SiO_2_ on InP(111)B substrates at 420 °C. Arrays with different pitches were investigated ranging from 100 to 700 nm. The growth time was 15 min, corresponding to a nominal planar growth of 58 nm thick InP for the data shown in Figure 7b. The In beam angle α was 45°. InAs markers were used to measure the growth kinetics of a thin InP NW having approximately uniform radius $R≅R_{0}=$ 12 nm from base to top. Although the authors claimed no significant pitch dependence of the NW length [16], the exponential curve based on Lambert re-emission without the shadowing effect shows a discrepancy for the longest growth time (dashed line in Figure 7b. The bold line in Figure 7b shows the fit obtained from Equations (10) to (13) for a hexagonal array with a pitch of 200 nm and gives a better quantitative agreement with the data compared to the original fit without shadowing.

## 8. Conclusions

To summarize, an analytic model has been developed for SAG of III-V NW arrays by MBE on reflecting substrates. The main ingredients of the model are (i) specular re-emission from the mask surface, (ii) shadowing effect under the assumption of an array of identical NWs, (iii) the absence of any surface growth on the substrate and desorption of group III atoms from the NW sidewalls or tops (with or without droplets), and (iv) an untapered NW shape. The assumption of a time-independent droplet volume for VLS NWs is not critical and can be refined in further calculations, although it should work perfectly well starting from a certain length due to a high NW aspect ratio. More complex tapered NW shapes will be considered elsewhere. An analytic expression has been derived for the total group III flux impinging the NW and for the shadowing length. It has been shown that (i) at a constant radius, NW length increases quite abruptly at the beginning of growth but in a more complex manner than exponential increase considered earlier, (ii) the NW volume scales exponentially with its length in the intermediate stage of growth, and (iii) in the asymptotic stage, the NW length and adius increases sublinearly with time or deposition thickness and finally reach a steady-state regime where the radius is constant and the NW elongation is linear in time. The model works equally well for VLS and non-VLS III-V NWs grown on reflecting substrates, as demonstrated by good fits obtained for different data. Finally, the model is quite general and may be useful for understanding and modeling MBE growth and morphology evolution of different NWs by SAG in MBE or CBE techniques.

## Figures and Tables

**Table 1:** Parameters used in Figure 4.

Pitch P (nm)	$L_{*}$ (nm)	$H_{*}$ (nm)	$H_{c}$ (nm)	B	C	$y_{1}$	$y_{2}$	ω
200	410	107	61.2	11.38	6.04	10.82	0.560	7.38
300	1194	172	61.5	24.12	13.60	23.55	0.575	18.26
400	2294	212	61.6	41.95	24.17	41.36	0.585	33.47

**Table 2:** Parameters used in Figure 5.

Pitch P (nm)	$L_{*}$ (nm)	$h_{*}$ (nm)	$L_{*}/h_{*}$	ε	${\left(L/H\right)}_{\infty }$	$R_{\infty }$
300	745	146	5.096	1.198	5.067	75.2
400	1240	137	9.058	0.8975	4.214	110
500	2180	154	14.156	0.7180	3.764	145

**Table 3:** Experimental data on the morphology of SAG InAs NWs [20].

	P= 250 nm		P= 500 nm		P= 1000 nm		P= 3000 nm	
Growth Time (min)	L (nm)	2R (nm)	L (nm)	2R (nm)	L (nm)	2R (nm)	L (nm)	2R (nm)
10	105.6	54.5	98.7	59.4	117.2	60.3	126.5	61.9
45	446.4	113.2	489	133.1	507.5	144.7	498.2	138.1
90	853.1	127.3	925.8	176.2	995.4	208.4	953.6	232.5
180	1311	180.3	1818	227.5	1883	316.9	1962	402.2
360	2168	191.9	3189	364.1	3524	530.5	3923	689.4

## Data Availability

Not applicable.
